# Perinatal Inflammation: Could Partial Blocking of Cell Adhesion Molecule Function Be a Solution?

**DOI:** 10.3390/children8050380

**Published:** 2021-05-12

**Authors:** Nikolaos Vrachnis, Dimitrios Zygouris, Dionysios Vrachnis, Nikolaos Roussos, Nikolaos Loukas, Nikolaos Antonakopoulos, Georgios Paltoglou, Stavroula Barbounaki, Georgios Valsamakis, Zoi Iliodromiti

**Affiliations:** 1 Third Department of Obstetrics and Gynecology, School of Medicine, National and Kapodistrian University of Athens, Attikon Hospital, 11526 Athens, Greece; antonakopoulos2002@yahoo.gr; 2 Vascular Biology, Molecular and Clinical Sciences Research Institute, St George’s University of London, London SW17 0RE, UK; 3 Research Centre in Obstetrics and Gynecology, Hellenic Society of Obstetric and Gynecologic Emergency, 11526 Athens, Greece; dzygour@med.uoa.gr (D.Z.); info@hsoge.gr (N.R.); 4 Department of Clinical Therapeutics, School of Medicine, National and Kapodistrian University of Athens, Alexandra Hospital, 11526 Athens, Greece; dionisisvrachnis@gmail.com; 5 Department of Gynecology, General Hospital of Athens “G. Gennimatas”, 11527 Athens, Greece; nloux13@hotmail.com; 6 Unit of Endocrinology, Diabetes Mellitus and Metabolism, School of Medicine, National and Kapodistrian University of Athens, Aretaieion Hospital, 11526 Athens, Greece; gpaltoglou@gmail.com (G.P.); gedvalsamakis@yahoo.com (G.V.); 7 Merchant Marine Academy of Aspropyrgos, 19300 Athens, Greece; sbarbounaki@yahoo.gr; 8 Department of Neonatology, School of Medicine, National and Kapodistrian University of Athens, Aretaieio Hospital, 11526 Athens, Greece; ziliodromiti@yahoo.gr

**Keywords:** prenatal, perinatal, neonatal inflammation, cell adhesion molecules, integrins, selectins, Ig superfamily

## Abstract

In spite of the great advances made in recent years in prenatal and perinatal medicine, inflammation can still frequently result in injury to vital organs and often constitutes a major cause of morbidity. It is today well established that in neonates—though vulnerability to infection among neonates is triggered by functional impairments in leukocyte adhesion—the decreased expression of cell adhesion molecules also decreases the inflammatory response. It is also clear that the cell adhesion molecules, namely, the integrins, selectins, and the immunoglobulin (Ig) gene super family, all play a crucial role in the inflammatory cascade. Thus, by consolidating our knowledge concerning the actions of these vital cell adhesion molecules during the prenatal period as well as regarding the genetic deficiencies of these molecules, notably leukocyte adhesion deficiency (LAD) I, II, and III, which can provoke severe clinical symptoms throughout the first year of life, it is anticipated that intervention involving blocking the function of cell adhesion molecules in neonatal leukocytes has the potential to constitute an effective therapeutic approach for inflammation. A promising perspective is the potential use of antibody therapy in preterm and term infants with perinatal inflammation and infection focusing on cases in which LAD is involved, while a further important scientific advance related to this issue could be the combination of small peptides aimed at the inhibition of cellular adhesion.

## 1. Introduction

Perinatal inflammation remains a significant cause of morbidity and mortality [1] despite recent developments in fetal medicine given that the inflammatory products can lead either to preterm birth or to injury to vital organs such as the lungs and brain [2]. Though the exact pathogenetic manner in which the sequence of events takes place resulting in tissue damage remains to date unknown, it is thought that cell adhesion molecules play a cornerstone role in inducing inflammatory response.

Adhesion has a fundamental role in the normal function of human cells, providing clues concerning migration as well as signals for growth differentiation and activation. Especially in leukocytes and platelets, adhesion amends their function under inflammatory conditions. In normal circumstances, these cells are in random contact with the endothelium and do not adhere to the vascular endothelium. They are, however, activated by inflammation and change their shape from spherical to flat [3]. In particular, neonatal neutrophils and monocytes are observed to display decreased migration and adhesion ability [4].

Several studies have conclusively demonstrated that when neonates suffer functional impairments in leukocyte adhesion, this results in vulnerability to infection [5], although the levels of adhesion molecules have also been used as a possible predictor of preterm labor in the presence of inflammation [1,6,7]. On the other hand, it is suggested that this functional impairment may play a protective role by decreasing the inflammatory response in the affected neonate.

The cell adhesion molecules that play a central role in leukocyte-endothelial interaction are classified into the following three categories: integrins, selectins, and the immunoglobulin (Ig) gene superfamily [8]. 

In this review, we summarize recent data on the role of adhesion molecules in the inflammatory cascade during the perinatal period and perform a more in-depth review of these proteins in order to identify their possible contribution to therapeutic interventions. 

The purpose of this review is to report and evaluate the recent data concerning cell adhesion molecules and perinatal inflammation and infection caused by leukocyte adhesion deficiency (LAD). We also present an overview of the expression and function of the most important cell adhesion molecules in the perinatal period and comment on the genetic deficiencies of these molecules.

## 2. Integrins Mediate Cell-Cell and Cell-Matrix Interactions

**Figure 1:**
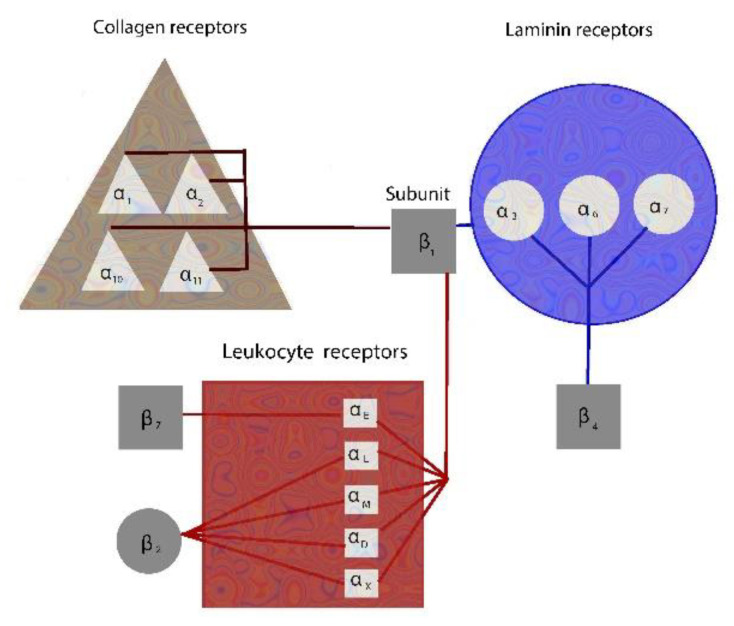
The integrin family. Integrins are heterodimers composed of 18 α subunits and 8 β subunits. β1 subunits participate in all three kinds of receptors: collagen, laminin, and leukocyte, while β2 and β7 participate in leukocyte receptors and β4 in laminin receptors.

Integrins are cell adhesion molecules that simultaneously mediate both cell to cell and cell to matrix signal transduction. The term integrin describes the receptors that “integrate” signals from the extracellular environment with intracellular molecules. Integrins are heterodimers, consisting of α and β subunits, which cross the membrane (transmembrane cell surface proteins) with most of their domain in the extracellular space. Until now, 18 α and 8 β chains have been characterized, forming a total of 24 distinct integrins that are obligate heterodimers composed of α and β subunits, participating in collagen, laminin, and leukocyte receptors (Figure 1). The initial production of α and β chains takes place in the cellular cytoplasm, both of these chains being necessary for complete β2 family differentiation, the β2 family acting as cell adhesion molecules. β2 and β7 integrins have been identified only in leukocytes, their cell surface co-receptors being members of the Ig gene superfamily. Integrins, which are mostly inactivated on leukocytes, are rapidly activated as a response to circulating inflammatory mediators and signal only after their activation. Macrophage-1 antigen (Mac-1) is a complement receptor that belongs to a family of cell surface co-receptors consisting of β2 integrins, while lymphocyte function-associated antigen 1 (LFA-1) is a heterodimeric glycoprotein which plays a key role in leukocyte emigration. Another key receptor and ligand of β2 integrins participating in leukocyte trafficking is RAGE (receptor for advanced glycation end products) [9].

Integrins on the intracellular side regulate the signaling complexes and the assemblies of the cytoskeleton proteins, while on the extracellular side, they bind to matrix macromolecules and counter-receptors [10]. Activation of integrins is completed by inside-out activation induced by ligand binding [11].

## 3. Selectins Mediate Leukocyte Interactions with Endothelium and Platelets

**Figure 2:**
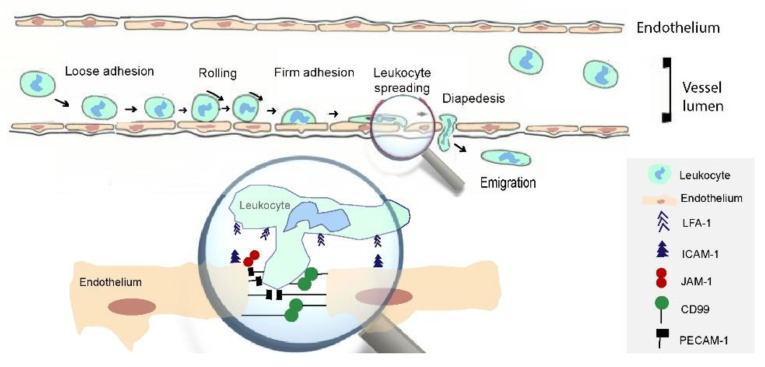
Mechanism of leukocyte transendothelial migration. Initially, there is loose adhesion, which gradually becomes firmer, resulting in diapedesis and emigration. Endothelial cell stimulation leads to ICAM-1 and JAM-1 up-regulation, thus enhancing the adhesion, while selectins enhance the attachment of the free-flowing leukocytes. ICAM-1: Intercellular Adhesion Molecule 1; JAM-1: junctional adhesion molecule-1; CD99: Cluster of differentiation 99; PECAM-1: Platelet endothelial cell adhesion molecule-1.

The selectin family is involved in the processes of acute and chronic inflammation by mediating interactions of leukocytes. The fact that selectins are able to connect with carbohydrate ligands is reflected in the name of the family, “selectin”, which contains the term “lectin” denoting specific carbohydrate-binding proteins that play a role in the phenomenon of cell-protein recognition. The selectin family, which is also known as CD62, includes three subcategories, namely L-selectin, E-selectin, and P-selectin. The main role of these cell adhesion molecules is attachment of the free-flowing leukocytes to the endothelium. The binding is accomplished through a calcium-dependent ligand [12] on the rolling leukocytes along the vascular endothelium. More specifically, L-selectin, which is found on the circulating lymphocytes, serves a vital purpose in the adhesion function of the peripheral lymph nodes. Moreover, they regulate neutrophil, eosinophil, and monocyte function. It was shown in animal models that inhibition of L-selectin molecules resulted in a reduced rolling velocity of the circulating leukocytes [13] (Figure 2).

E-selectin is mostly expressed on endothelial cells and their production is induced by inflammatory mediators such as Interleukin-1 (IL-1), Interleukin-6 (IL-6), Tumor necrosis factora (TNF-a), and Tumor necrosis factor-b (TNF-b). Maximal expression is achieved in 6 hours, and within 24 hours they once again return to basal levels [14]. 

The third subcategory is P-selectin, which is produced in the endothelial cells that are sorted into secretory granules until they are mobilized on the cellular surface. The mobilization of P-selectin is mostly induced by inflammatory cytokines and reactive oxygen species (ROS), resulting in adhesion between platelets, leukocytes, and endothelial cells [15].

Intercellular Adhesion Molecule-1 (ICAM-1) is a widely investigated adhesion molecule known for its importance in stabilizing cell-cell interactions and facilitating leukocyte endothelial transmigration. Although prediction of the severity of infection has until now been possible only via soluble ICAM-1 [16], it is proposed that the soluble cell adhesion molecules P-selectin, E-selectin, and Vascular cell adhesion molecule-1 (VCAM-1) could potentially be used as a predictor of the clinical outcome in infants with inflammation. However, to date, a clearly significant correlation between soluble selectins and inflammation in infants has not been firmly established. 

Other soluble adhesion molecules interacting with selectins have also been reported to increase in obstetrical conditions such as preeclampsia, small for gestational age (SGA) and IUGR fetuses, pyelonephritis, preterm labor with intact membranes (PTL), and preterm rupture of membranes (PROM) [17,18]. Levels of ICAM-1, VCAM-1, and E-selectin were significantly higher in women with preeclampsia and IUGR (intrauterine growth restriction) [19]. Statistically significant correlations between increased serum levels of ICAM-1, VCAM-1, L- selectin, and P- selectin in women with preterm labor have also been reported [20,21].

## 4. Role of Immunoglobulin Gene (Ig) Super Family in Leukocyte Adherence

**Figure 3:**
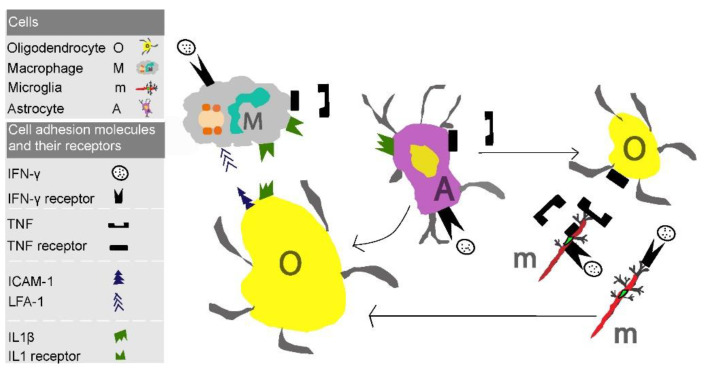
In the model of oligodendrocyte cell damage and death, Ig superfamily have a pivotal role leading in neurodevelopment problems. Macrophages and microglia adhere to oligodendrocytes through ICAM1 and LFA-1. Additionally, high levels of inflammatory cytokines shown in the figure enhance this interaction, thus accelerating cell damage and death. IFN-γ: Interferon gamma; TNF: Tumor necrosis factor; IL-1β: Interleukin 1β; ICAM-1: Intercellular Adhesion Molecule 1.

Cell adhesion molecules of the Ig super family are crucial for leukocyte-endothelial and leukocyte-leukocyte adherence. Apart from their role in leukocyte transendothelial migration, they also play an essential part in oligodendrocyte cell damage and death, contributing significantly to adverse perinatal outcomes and neurodevelopmental disability (Figure 3).

The most important Ig superfamily ligands are ICAM-1, ICAM-2, VCAM-1 and Platelet endothelial cell adhesion molecule-1 (PECAM-1), all expressed on leukocytes [20,21]. ICAM-1 has a structure composed of five Ig domains and is mostly expressed on endothelial cells and leukocytes. ICAM-1 expression is rapidly stimulated by inflammatory cytokines [22], such as IL-1, IL-6, and TNF-a, as well as by several cell types including leucocytes, fibroblasts, and endothelial cells and, less commonly, on hepatocytes and dendritic cells [23].

VCAM-1, consists of seven Ig domains and is expressed mainly on nonvascular cells, while it does not exist on endothelial cells [24]. VCAM-1 expression is a result of an inflammatory response, stimulated by IL-1, IL-6, reactive oxygen species (ROS), high glucose concentration, and TNF-a [2,25]. VCAM-1 plays a major role in migration and adherence of lymphocytes, reaching maximal levels in 12 hours after the initial induction [26].

PECAM-1 contains six Ig domains and is found on lymphocytes, neutrophils, monocytes, and platelets. Its highest expression is on endothelial cells, contributing to endothelial-platelet adhesion and transendothelial migration of circulating leukocytes [27].

## 5. Integrin Function in the Perinatal Period

(a) β-2 subfamily integrins

It is well known that inflammatory cytokines cause neutrophil activation both in adults and in neonates, although neonatal neutrophils do not have the same ability to adhere to cell matrix proteins as do adult neutrophils [28]. On adult neutrophils, the level of macrophage-1 antigen (Mac-1) is very low when neutrophils are inactivated, although Mac-1 antigen levels increase rapidly after cell activation [29]. Several studies have shown that, by contrast, neonatal neutrophils do not react in the same way to the inflammatory mediator [29,30]. After in vitro stimulation, the expression of Mac-1 was found to be considerably lower in comparison to adult neutrophils, while the total expression of Mac-1 was half that of adult cells [31]. In premature neonates, Mac-1 expression is even lower [32], at about one-third of adult expression; this happens at both stages, before and after stimulation, which clearly reflects their decreased ability to react to inflammatory cytokines. Mac-1 expression finally reaches adult levels after the first year of life [33]. In term infants with inflammation, Mac-1 expression was observed to be significantly increased on circulating neutrophils as compared to healthy neonates [22]. Moreover, increased levels of Mac-1 expression in leukocytes in infants were shown to be predictive of later development of sepsis [34] and, in this context, were also strongly correlated with the need for mechanical ventilation [35].

Other studies in neonates demonstrate decreased transmigration capacity in neonatal neutrophils, which is about 50% compared with adult neutrophils [29]. This can be explained by low lymphocyte function-associated antigen 1 (LFA-1) expression in neonatal cells, which is even lower in preterm infants before the 35th week of gestation [36]. Therefore, the reduced expression and function of Mac-1 and LFA-1 most likely account for the delayed reaction in preterm and term infants (this induced by chemotactic stimulation), which seems to be a consequence of impaired neonatal neutrophil motility. Recently, research interest has focused on receptor of advanced glycation end products (RAGE), with investigations into whether it participates in leukocyte adhesion. The results of these studies suggest that RAGE impair leukocyte adhesion in very premature infants, despite high expression, their expression possibly differing from function [37].

(b) β-1 and β-3 subfamily integrins

Levels of β1 and β3 integrins in neonatal neutrophils are similar to those in adult neutrophils [38]. However, neonatal neutrophils display impaired emigration capacity that can be attributed to reduced β2 integrin function. When neonatal neutrophils are able to traverse the endothelial barrier, this denotes that they have attained adult neutrophil function. Meanwhile, it has been established that β1-integrin is necessary in fetal lung development and participates in the angiogenesis of the eye [39].

In contrast, the proportion of NK cells (4% vs. 10.5%) and activated T-cells (CD3+CD25+, 7.0% vs. 15%) is decreased in neonatal blood compared to that in adults [40], while there are also studies demonstrating that lymphocytes remain ‘naïve’ in neonates: this is because they are still at the stage between maturity and activation, having not yet encountered their corresponding antigen [41]. The integrin subfamily β-3 mediates the adhesion of platelets with fibrinogen and other ligands. Since normal production and function of β1 integrins is necessary for adhesion of T-lymphocytes to the extracellular matrix, the lower expression of β1 integrins in neonates could be an explanation for this ‘naive’ state. Moreover, the lack of the appropriate chemokine receptor in neonatal lymphocytes [42] possibly accounts for their impaired inflammatory and chemotactic response.

Platelet adhesion is mediated by integrin subfamily β-3 together with fibrinogen and other ligands, while in inflammation and thrombosis, interactions between the injured tissue and circulating cells are mediated by integrins β 1-3 [38].

## 6. Selectin Function in the Perinatal Period

Neonatal neutrophils and eosinophils in preterm and term infants have been found to express decreased L-selectin levels [43], although fetal neutrophils and eosinophils have the same levels of L-selectin as do adults [44]. This can be explained by the apoptosis of L-selectin from the cellular surface in preterm and term infants [45]. It nevertheless remains unclear whether the condition involving decreased L-selectin levels arises within the course of maturational development or through activation of in-utero inflammation. In addition, soluble L-selectin, which peels off the cell surface, is also decreased compared to adult levels [46].

In cases of infants with a bacterial infection, L-selectin expression was seen to be decreased in neonatal neutrophils and monocytes when these infants were compared with healthy infants [47]. Animal studies with inhibition of L-selectin by IL-1b revealed a significant impact on leukocyte emigration in neonates compared to adults [48]. Furthermore, soluble L-selectin was also observed to be higher in the bronchoalveolar fluid of infants that developed chronic lung disease (CLD) [49].

Concerning P-selectins, to date, there are a few data demonstrating decreased up-regulation of P-selectin in neonatal platelets of healthy term neonates compared to those in adults [50]. In animal studies, fetal inflammation resulted in P-selectin deficiency [51] as well as in diminished leukocyte influx in excisional wounds compared to adult animals. Moreover, in human studies, it was revealed that P-selectin expression in endothelial cells increases with gestational age [52]. Soluble P-selectin and soluble E-selectin were significantly increased in the plasma of preterm infants, with no difference in levels observed to those in infants who developed CLD [53].

## 7. Ig Superfamily Function in the Perinatal Period

Increased levels of ICAM-1 in maternal, fetal, and intra-amniotic compartments contribute to preterm birth and adverse pregnancy outcomes [7,54]. A study on very premature infants born before 28 weeks of gestation showed an association of increased levels of ICAM-1 in blood with retinopathy of prematurity [55]. In infants born before 28 weeks, postnatal inflammation 3–4 weeks later showed that increased ICAM-1 levels in blood spots were associated with low psychomotor development index (PDI) < 55 [56].

Moreover ICAM-1 levels in plasma were associated with bronchopulmonary dysplasia (BPD) severity and outcome [57], while also serum levels were an indicator of perinatal asphyxia [58]. The same findings were previously reported in immature baboons in tracheal aspirate fluids [59] and in lungs of neonatal mice, where inhaled nitric oxide improved the hyperoxia-induced up-regulated ICAM-1 levels [60]. 

In premature infants, especially those born <28 weeks of gestation, PECAM-1 in blood is decreased, this accompanied by a reduction in the number of capillaries and small arteries, leading to BPD [61].

## 8. Genetic Mutations Result in Leukocyte Adhesion Deficiency

Four major genetic deficiencies have been diagnosed in the neonatal period, namely, leukocyte adhesion deficiency (LAD) I, II, III, and IV. LAD I is characterized by recurrent infections of soft tissues, membranes, and lungs, these presenting as pneumonitis, esophagitis, laryngitis, omphalitis, delayed separation of the umbilical cord, and necrotic skin lesions [62,63,64,65]. In adult humans, diagnosis of this deficiency is made using flow cytometry in order to determine the immunophenotype of the leukocytes. Prenatally, it is performed with flow cytometry in fetal blood retrieved by cordocentesis during the 18–20th weeks of gestation [66]. Since the chief characteristic among these patients is recurrent viral infections, this implies that the major deficiency is in the function of neutrophils, followed by T-lymphocyte deficiency. The skin lesions start as small erythymatosous and often progress to necrotic ulcers, which are very difficult to treat. Periodontitis is also common among these patients and there are also reported cases of perianal abscess that can even lead to peritonitis or brain abscesses [52,67,68].

To date, many animal and human studies have revealed that the cause of this deficiency is mutations in the gene encoding the β chain, this damage resulting in absolute or partial deficiency of the β-2 integrin family [69,70,71]. The above data thus establish the cause of the immunologic defects in these human neonates while accounting for the variable clinical features that they manifest.

LAD II is characterized by damage to the endogenous fucose metabolism. As neutrophils are fucose-containing cells, they are significantly affected, losing their ability to bind P- and E-selectins, which are normally expressed in these patients. The clinical features are similar to those of LAD I, characterized by recurrent infections, though milder neutrophilia than in LAD I, as well as mental and growth retardation due to the metabolic consequences of fucose-deficiency [72,73].

Another leukocyte adhesion molecule deficiency is LAD III, which is mainly characterized by mutations of FERMT 3 (fermitin family homolog 3), which encodes kindlin 3, a protein that activates β2 integrin. Besides the failure of lymphocytes to bind to integrin ligands, platelets also demonstrate inability to respond to integrin [74]. This disease clinically presents with epistaxis, purpura, and bleeding as a result of an either qualitative or quantitative defect on platelets and recurrent infections without abscesses or leykocytosis. There are also reported cases of osteopetrosis due to the impact of gene mutations on osteoclast function [74,75,76]. Whereas the expression of LFA-1 and Mac-1 remains normal, these ligands function defectively [77]. Studies have recently revealed that although there is activation by inflammatory chemokines, integrins fail to aggregate the platelets due to their functionally defective β1, β2, or β3 chains [78].

Finally, LAD IV is a deficiency characterized by effects of cystic fibrosis transmembrane conductance regulator (CFTR) defect on integrin activation. Patients with LAD IV present with progressively worsening lung infections, chronic pancreatitis, and chronic rhinosinusitis [79,80].

## 9. Future Perspectives

Infants with LAD I disorder may exhibit severe clinical symptoms during the first 12 months of life [63]. To date, given that there is no established treatment for these patients, even though the diagnosis is clear, they remain under close surveillance to monitor for any signs of inflammation. Antibiotic therapy is used as a first-line treatment in mild cases, which may subsequently be followed by granulocyte transfusion [78]. In more severe forms of LAD I, bone marrow transplantation has also been used with good outcomes, as well as stem cell transplantation, despite the latter’s high complication rate [81,82]. Treatment with immunoglobulin for non-healing ulcers has also been suggested, this additionally providing better control of severe infections [83]. Other treatments that have been applied are interleukin-12 combined with ustekinumab, an antibody against interleukin-23. The latter treatment was administered to a patient with severe periodontitis and an intractable, deep, non-healing sacral wound, while in animal studies, it protected mice from lethal *Citrobacter rodentium*-induced colitis [84,85]. Fuller understanding of the molecular basis of this deficiency offers a promising perspective for introduction of genes into the β2 chain of human hematopoietic stem cells, although until now the only report of successful treatment of this type has been in canine and other animal models [83,84,85]. Recently, successful treatment in a girl with LAD-1 with anti-tumor necrosis factor-a (TNF-a) was reported [86].

Standard dexamethasone treatment has been used in several studies in order to decrease the concentration of plasma soluble E-selectins while concurrently increasing soluble L-selectin [87,88]. The fact that the medication moreover modulates inflammatory response in preterm infants [89,90,91] through modulating circulating cell adhesion molecules implies that this may constitute a possible future clinical intervention [92]. However, further investigation is necessary into the use of cortisone, statins, and other drugs for the modulation of cell adhesion molecules.

Another intervention that shows promise is the use of antibodies for an anti-adhesion targeted therapy in preterm and term infants with perinatal inflammation. To date, antibody therapy has been widely used in patients after coronary angioplasty for prevention of reinfarction [41] and in preclinical studies in patients with Crohn’s disease, psoriasis, and multiple sclerosis. In adults, a number of integrins (αvβ3 and β7 integrins (α4β7 and αEβ7 integrins)) have been targeted with monoclonal antibodies [93,94], while more specific integrin antagonists have been used in patients after percutaneous angioplasty. Moreover, natalizumab, a monoclonal antibody against integrins, has been used for the treatment of multiple sclerosis, however, with serious side effects, as it caused progressive multifocal leuko-encephalopathy (PML). On the other hand, vedolizumab, a new antibody against integrins, is currently used for multiple sclerosis without causing PML, as well as in the treatment of inflammatory bowel disease.

A further step towards establishing such a therapy could be the development of small peptides aimed at inhibition of cellular adhesion. Lovastatin is an anti-adhesive drug that has already been used for the inhibition of ICAM-1 adhesion ability [95]. Similar drugs may be developed in future for the inhibition of inflammation-induced injury in neonates. Targeted antibody inhibition of cell adhesion molecules will diminish inflammation and bone loss and may be preferable to glucocorticoid and other immunosuppressive drugs.

Furthermore, the use of anti-adhesion therapy can be enhanced by combining other therapeutic interventions. For example, in preterm infants with respiratory distress syndrome, mechanical ventilation is necessary for life support, although it simultaneously causes neutrophil activation [96]. A strategy that shows potential in these cases appears to be the use of combined anti-adhesion therapy and mechanical ventilation in order to achieve an optimal clinical outcome [97].

## 10. Conclusions

This article comprises a narrative review of the recent data concerning cell adhesion molecules, with particular focus on infection and inflammation in the perinatal period. Selectins, integrins, and the Ig superfamily play a crucial role in the inflammatory cascade, and we have herein presented the recent experimental and clinical data relating to the molecular basis of adhesion in perinatal inflammation. It is evident that more studies are necessary to clarify the exact molecular mechanism in order to develop new strategies for improvement of perinatal outcomes. Finally, interventions consisting in blocking the function of cell adhesion molecules in neonatal leukocytes and other blood cell lines are highly likely to comprise an effective therapeutic approach. Further clinical studies are in every case required before the introduction of new drugs and antibody therapies into the standard treatment for perinatal inflammation.

## References

[1] Vrachnis N., Vitoratos N., Iliodromiti Z., Deligeoroglou E., Creatsas G. (2010). Intrauterine inflammation and preterm delivery. Ann. N. Y. Acad. Sci..

[2] Iliodromiti Z., Zygouris D., Sifakis S., Pappa K.I., Tsikouras P., Salakos N., Daniilidis A., Siristadidis C., Vrachnis N. (2013). Acute lung injury in preterm fetuses and neonates: Mechanisms and molecular pathways. J. Matern. Fetal Neonatal Med..

[3] Anderson D.C., Hughes B.J., Smith C.W. (1981). Abnormal mobility of neonatal polymorphonuclear leukocytes. Relationship to impaired redistribution of surface adhesion sites by chemotactic factor or colchicine. J. Clin. Investig..

[4] Iliodromiti Z., Iliodromiti Z., Anastasiadis A., Varras M., Pappa K.I., Siristatidis C., Bakoulas V., Mastorakos G., Vrachnis N. (2013). Monocyte function in the fetus and the preterm neonate: Immaturity combined with functional impairment. Mediat. Inflamm..

[5] Carr R. (2000). Neutrophil production and function in newborn infants. Br. J. Haematol..

[6] Vrachnis N., Malamitsi-Puchner A., Samoli E., Botsis D., Iliodromiti Z., Baka S., Hassiakos D., Creatsas G. (2006). Elevated mid-trimester amniotic fluid ADAM-8 concentrations as a potential risk factor for preterm delivery. J. Soc. Gynecol. Investig..

[7] Malamitsi-Puchner A., Vrachnis N., Samoli E., Baka S., Alexandrakis G., Puchner K.P., Iliodromiti Z., Hassiakos D. (2006). Investigation of midtrimester amniotic fluid factors as potential predictors of term and preterm deliveries. Mediat. Inflamm..

[8] Smith C.W. (2008). 3. Adhesion molecules and receptors. J. Allergy Clin. Immunol..

[9] Zarbock A., Ley K. (2009). Neutrophil adhesion and activation under flow. Microcirculation.

[10] Giancotti F.G., Ruoslahti E. (1999). Integrin signaling. Science.

[11] Mokhtar D.M., Abdelhafez E.A. (2021). An overview of the structural and functional aspects of immune cells in teleosts. Histol. Histopathol..

[12] Sperandio M. (2006). Selectins and glycosyltransferases in leukocyte rolling in vivo. FEBS J..

[13] Landgraf M.A., Martinez L.L., Rastelli V.M., Franco Mdo C., Soto-Suazo M., Tostes Rde C., Carvalho M.H., Nigro D., Fortes Z.B. (2005). Intrauterine undernutrition in rats interferes with leukocyte migration, decreasing adhesion molecule expression in leukocytes and endothelial cells. J. Nutr..

[14] Leeuwenberg J.F., Smeets E.F., Neefjes J.J., Shaffer M.A., Cinek T., Jeunhomme T.M., Ahern T.J., Buurman W.A. (1992). E-selectin and intercellular adhesion molecule-1 are released by activated human endothelial cells in vitro. Immunology.

[15] Mittal M., Siddiqui M.R., Tran K., Reddy S.P., Malik A.B. (2014). Reactive oxygen species in inflammation and tissue injury. Antioxid. Redox Signal..

[16] Figueras-Aloy J., Gomez-Lopez L., Rodriguez-Miguelez J.M., Salvia-Roiges M.D., Jordan-Garcia I., Ferrer-Codina I., Carbonell-Estrany X., Jimenez-Gonzalez R. (2007). Serum soluble ICAM-1, VCAM-1, L-selectin, and P-selectin levels as markers of infection and their relation to clinical severity in neonatal sepsis. Am. J. Perinatol..

[17] Vrachnis N., Karavolos S., Iliodromiti Z., Sifakis S., Siristatidis C., Mastorakos G., Creatsas G. (2012). Review: Impact of mediators present in amniotic fluid on preterm labour. In Vivo.

[18] Docheva N., Romero R., Chaemsaithong P., Tarca A.L., Bhatti G., Pacora P., Panaitescu B., Chaiyasit N., Chaiworapongsa T., Maymon E. (2019). The profiles of soluble adhesion molecules in the “great obstetrical syndromes”. J. Matern. Fetal Neonatal Med..

[19] Coata G., Pennacchi L., Bini V., Liotta L., Di Renzo G.C. (2002). Soluble adhesion molecules: Marker of pre-eclampsia and intrauterine growth restriction. J. Matern. Fetal Neonatal Med..

[20] Huang M.T., Larbi K.Y., Scheiermann C., Woodfin A., Gerwin N., Haskard D.O., Nourshargh S. (2006). ICAM-2 mediates neutrophil transmigration in vivo: Evidence for stimulus specificity and a role in PECAM-1-independent transmigration. Blood.

[21] Woodfin A., Voisin M.B., Nourshargh S. (2007). PECAM-1: A multi-functional molecule in inflammation and vascular biology. Arterioscler. Thromb. Vasc. Biol..

[22] Malamitsi-Puchner A., Vrachnis N., Samoli E., Baka S., Iliodromiti Z., Puchner K.P., Malligianis P., Hassiakos D. (2006). Possible early prediction of preterm birth by determination of novel proinflammatory factors in midtrimester amniotic fluid. Ann. N. Y. Acad. Sci..

[23] Vestweber D., Winderlich M., Cagna G., Nottebaum A.F. (2009). Cell adhesion dynamics at endothelial junctions: VE-cadherin as a major player. Trends Cell Biol..

[24] Jones E.Y., Harlos K., Bottomley M.J., Robinson R.C., Driscoll P.C., Edwards R.M., Clements J.M., Dudgeon T.J., Stuart D.I. (1995). Crystal structure of an integrin-binding fragment of vascular cell adhesion molecule-1 at 1.8 A resolution. Nature.

[25] Park E.J., Myint P.K., Ito A., Appiah M.G., Darkwah S., Kawamoto E., Shimaoka M. (2020). Integrin-Ligand Interactions in Inflammation, Cancer, and Metabolic Disease: Insights into the Multifaceted Roles of an Emerging Ligand Irisin. Front. Cell Dev. Biol..

[26] Cook-Mills J.M. (2002). VCAM-1 signals during lymphocyte migration: Role of reactive oxygen species. Mol. Immunol..

[27] Privratsky J.R., Newman D.K., Newman P.J. (2010). PECAM-1: Conflicts of interest in inflammation. Life Sci..

[28] Raymond S.L., Mathias B.J., Murphy T.J., Rincon J.C., Lopez M.C., Ungaro R., Ellett F., Jorgensen J., Wynn J.L., Baker H.V. (2017). Neutrophil chemotaxis and transcriptomics in term and preterm neonates. Transl. Res..

[29] Anderson D.C., Rothlein R., Marlin S.D., Krater S.S., Smith C.W. (1990). Impaired transendothelial migration by neonatal neutrophils: Abnormalities of Mac-1 (CD11b/CD18)-dependent adherence reactions. Blood.

[30] Torok C., Lundahl J., Hed J., Lagercrantz H. (1993). Diversity in regulation of adhesion molecules (Mac-1 and L-selectin) in monocytes and neutrophils from neonates and adults. Arch. Dis. Child..

[31] Abughali N., Berger M., Tosi M.F. (1994). Deficient total cell content of CR3 (CD11b) in neonatal neutrophils. Blood.

[32] Nupponen I., Pesonen E., Andersson S., Makela A., Turunen R., Kautiainen H., Repo H. (2002). Neutrophil activation in preterm infants who have respiratory distress syndrome. Pediatrics.

[33] Storm S.W., Mariscalco M.M., Tosi M.F. (2008). Postnatal maturation of total cell content and up-regulated surface expression of Mac-1 (CD11b/CD18) in polymorphonuclear leukocytes of human infants. J. Leukoc. Biol..

[34] Weinschenk N.P., Farina A., Bianchi D.W. (2000). Premature infants respond to early-onset and late-onset sepsis with leukocyte activation. J. Pediatr..

[35] Sarafidis K., Drossou-Agakidou V., Kanakoudi-Tsakalidou F., Taparkou A., Tsakalidis C., Tsandali C., Kremenopoulos G. (2001). Evidence of early systemic activation and transendothelial migration of neutrophils in neonates with severe respiratory distress syndrome. Pediatr. Pulmonol..

[36] McEvoy L.T., Zakem-Cloud H., Tosi M.F. (1996). Total cell content of CR3 (CD11b/CD18) and LFA-1 (CD11a/CD18) in neonatal neutrophils: Relationship to gestational age. Blood.

[37] Buschmann K., Tschada R., Metzger M.S., Braach N., Kuss N., Hudalla H., Poeschl J., Frommhold D. (2014). RAGE controls leukocyte adhesion in preterm and term infants. BMC Immunol..

[38] Gonzalez A.L., El-Bjeirami W., West J.L., McIntire L.V., Smith C.W. (2007). Transendothelial migration enhances integrin-dependent human neutrophil chemokinesis. J. Leukoc. Biol..

[39] Douglass S., Goyal A., Iozzo R.V. (2015). The role of perlecan and endorepellin in the control of tumor angiogenesis and endothelial cell autophagy. Connect. Tissue Res..

[40] O’Gorman M.R., Millard D.D., Lowder J.N., Yogev R. (1998). Lymphocyte subpopulations in healthy 1-3-day-old infants. Cytometry.

[41] Pilarski L.M., Yacyshyn B.R., Jensen G.S., Pruski E., Pabst H.F. (1991). Beta 1 integrin (CD29) expression on human postnatal T cell subsets defined by selective CD45 isoform expression. J. Immunol..

[42] Sato K., Kawasaki H., Nagayama H., Enomoto M., Morimoto C., Tadokoro K., Juji T., Takahashi T. (2001). Chemokine receptor expressions and responsiveness of cord blood T cells. J. Immunol..

[43] Koenig J.M., Stegner J.J., Schmeck A.C., Saxonhouse M.A., Kenigsberg L.E. (2005). Neonatal neutrophils with prolonged survival exhibit enhanced inflammatory and cytotoxic responsiveness. Pediatr. Res..

[44] Kim S.K., Keeney S.E., Alpard S.K., Schmalstieg F.C. (2003). Comparison of L-selectin and CD11b on neutrophils of adults and neonates during the first month of life. Pediatr. Res..

[45] Sundqvist M., Osla V., Jacobsson B., Rudin A., Savman K., Karlsson A. (2013). Cord blood neutrophils display a galectin-3 responsive phenotype accentuated by vaginal delivery. BMC Pediatr..

[46] Lawrence S.M., Corriden R., Nizet V. (2017). Age-Appropriate Functions and Dysfunctions of the Neonatal Neutrophil. Front. Pediatr..

[47] Buhrer C., Graulich J., Stibenz D., Dudenhausen J.W., Obladen M. (1994). L-selectin is down-regulated in umbilical cord blood granulocytes and monocytes of newborn infants with acute bacterial infection. Pediatr. Res..

[48] Mariscalco M.M., Vergara W., Mei J., Smith E.O., Smith C.W. (2002). Mechanisms of decreased leukocyte localization in the developing host. Am. J. Physiol. Heart Circ. Physiol..

[49] Orwoll B.E., Sapru A. (2016). Biomarkers in Pediatric ARDS: Future Directions. Front. Pediatr..

[50] Rajasekhar D., Kestin A.S., Bednarek F.J., Ellis P.A., Barnard M.R., Michelson A.D. (1994). Neonatal platelets are less reactive than adult platelets to physiological agonists in whole blood. Thromb. Haemost..

[51] Olutoye O.O., Zhu X., Cass D.L., Smith C.W. (2005). Neutrophil recruitment by fetal porcine endothelial cells: Implications in scarless fetal wound healing. Pediatr. Res..

[52] Nussbaum C., Gloning A., Pruenster M., Frommhold D., Bierschenk S., Genzel-Boroviczeny O., von Andrian U.H., Quackenbush E., Sperandio M. (2013). Neutrophil and endothelial adhesive function during human fetal ontogeny. J. Leukoc. Biol..

[53] Ramsay P.L., O’Brian Smith E., Hegemier S., Welty S.E. (1998). Early clinical markers for the development of bronchopulmonary dysplasia: Soluble E-Selectin and ICAM-1. Pediatrics.

[54] Brou L., Almli L.M., Pearce B.D., Bhat G., Drobek C.O., Fortunato S., Menon R. (2012). Dysregulated biomarkers induce distinct pathways in preterm birth. BJOG.

[55] Holm M., Morken T.S., Fichorova R.N., VanderVeen D.K., Allred E.N., Dammann O., Leviton A. (2017). Neonatology Elgan Study Ophthalmology Committees. Systemic Inflammation-Associated Proteins and Retinopathy of Prematurity in Infants Born Before the 28th Week of Gestation. Investig. Ophthalmol. Vis. Sci..

[56] Leviton A., Allred E.N., Fichorova R.N., Kuban K.C., Michael O’Shea T., Dammann O., Elgan Study Investigators (2016). Systemic inflammation on postnatal days 21 and 28 and indicators of brain dysfunction 2 years later among children born before the 28th week of gestation. Early Hum. Dev..

[57] Sahni M., Yeboah B., Das P., Shah D., Ponnalagu D., Singh H., Nelin L.D., Bhandari V. (2020). Novel biomarkers of bronchopulmonary dysplasia and bronchopulmonary dysplasia-associated pulmonary hypertension. J. Perinatol..

[58] Huseynova S., Panakhova N., Orujova P., Hasanov S., Guliyev M., Orujov A. (2014). Elevated levels of serum sICAM-1 in asphyxiated low birth weight newborns. Sci. Rep..

[59] Coalson J.J., Winter V.T., Siler-Khodr T., Yoder B.A. (1999). Neonatal chronic lung disease in extremely immature baboons. Am. J. Respir. Crit. Care Med..

[60] Rose M.J., Stenger M.R., Joshi M.S., Welty S.E., Bauer J.A., Nelin L.D. (2010). Inhaled nitric oxide decreases leukocyte trafficking in the neonatal mouse lung during exposure to >95% oxygen. Pediatr. Res..

[61] Thebaud B., Ladha F., Michelakis E.D., Sawicka M., Thurston G., Eaton F., Hashimoto K., Harry G., Haromy A., Korbutt G. (2005). Vascular endothelial growth factor gene therapy increases survival, promotes lung angiogenesis, and prevents alveolar damage in hyperoxia-induced lung injury: Evidence that angiogenesis participates in alveolarization. Circulation.

[62] Anderson D.C., Schmalsteig F.C., Finegold M.J., Hughes B.J., Rothlein R., Miller L.J., Kohl S., Tosi M.F., Jacobs R.L., Waldrop T.C. (1985). The severe and moderate phenotypes of heritable Mac-1, LFA-1 deficiency: Their quantitative definition and relation to leukocyte dysfunction and clinical features. J. Infect. Dis..

[63] Rivera-Matos I.R., Rakita R.M., Mariscalco M.M., Elder F.F., Dreyer S.A., Cleary T.G. (1995). Leukocyte adhesion deficiency mimicking Hirschsprung disease. J. Pediatr..

[64] Sivathanu S., Sampath S., Sridhar I. (2016). Case 1: Recurrent Omphalitis and Nonhealing Ulcers in a 7-month-old Girl. Pediatr. Rev..

[65] Webber E.C., Church J., Rand T.H., Shah A.J. (2007). Leukocyte adhesion deficiency in a female patient without delayed umbilical cord separation. J. Paediatr. Child Health.

[66] Mishra A., Gupta M., Dalvi A., Ghosh K., Madkaikar M. (2014). Rapid Flow cytometric prenatal diagnosis of primary immunodeficiency (PID) disorders. J. Clin. Immunol..

[67] Silva L.M., Brenchley L., Moutsopoulos N.M. (2019). Primary immunodeficiencies reveal the essential role of tissue neutrophils in periodontitis. Immunol. Rev..

[68] Kumar A., Gupta A., Rawat A., Ahuja C., Suri D., Singh S. (2016). Brain Abscess in a Child with Leukocyte Adhesion Defect: An Unusual Association. J. Clin. Immunol..

[69] Hynes R.O. (1996). Targeted mutations in cell adhesion genes: What have we learned from them?. Dev. Biol..

[70] Etzioni A., Doerschuk C.M., Harlan J.M. (1999). Of man and mouse: Leukocyte and endothelial adhesion molecule deficiencies. Blood.

[71] Shaw J.M., Al-Shamkhani A., Boxer L.A., Buckley C.D., Dodds A.W., Klein N., Nolan S.M., Roberts I., Roos D., Scarth S.L. (2001). Characterization of four CD18 mutants in leucocyte adhesion deficient (LAD) patients with differential capacities to support expression and function of the CD11/CD18 integrins LFA-1, Mac-1 and p150,95. Clin. Exp. Immunol..

[72] von Andrian U.H., Berger E.M., Ramezani L., Chambers J.D., Ochs H.D., Harlan J.M., Paulson J.C., Etzioni A., Arfors K.E. (1993). In vivo behavior of neutrophils from two patients with distinct inherited leukocyte adhesion deficiency syndromes. J. Clin. Investig..

[73] Etzioni A., Frydman M., Pollack S., Avidor I., Phillips M.L., Paulson J.C., Gershoni-Baruch R. (1992). Brief report: Recurrent severe infections caused by a novel leukocyte adhesion deficiency. N. Engl. J. Med..

[74] Crazzolara R., Maurer K., Schulze H., Zieger B., Zustin J., Schulz A.S. (2015). A new mutation in the KINDLIN-3 gene ablates integrin-dependent leukocyte, platelet, and osteoclast function in a patient with leukocyte adhesion deficiency-III. Pediatr. Blood Cancer.

[75] Harris E.S., Shigeoka A.O., Li W., Adams R.H., Prescott S.M., McIntyre T.M., Zimmerman G.A., Lorant D.E. (2001). A novel syndrome of variant leukocyte adhesion deficiency involving defects in adhesion mediated by beta1 and beta2 integrins. Blood.

[76] Fagerholm E.D., Moran R.J., Violante I.R., Leech R., Friston K.J. (2020). Dynamic causal modelling of phase-amplitude interactions. NeuroImage.

[77] Alon R., Aker M., Feigelson S., Sokolovsky-Eisenberg M., Staunton D.E., Cinamon G., Grabovsky V., Shamri R., Etzioni A. (2003). A novel genetic leukocyte adhesion deficiency in subsecond triggering of integrin avidity by endothelial chemokines results in impaired leukocyte arrest on vascular endothelium under shear flow. Blood.

[78] Etzioni A. (2009). Genetic etiologies of leukocyte adhesion defects. Curr. Opin. Immunol..

[79] Fan Z., Ley K. (2016). Leukocyte Adhesion Deficiency IV. Monocyte Integrin Activation Deficiency in Cystic Fibrosis. Am. J. Respir. Crit. Care Med..

[80] Sorio C., Montresor A., Bolomini-Vittori M., Caldrer S., Rossi B., Dusi S., Angiari S., Johansson J.E., Vezzalini M., Leal T. (2016). Mutations of Cystic Fibrosis Transmembrane Conductance Regulator Gene Cause a Monocyte-Selective Adhesion Deficiency. Am. J. Respir. Crit. Care Med..

[81] Qian X., Wang P., Wang H., Jiang W., Sun J., Wang X., Zhai X. (2020). Successful umbilical cord blood transplantation in children with leukocyte adhesion deficiency type I. Transl. Pediatr..

[82] Thomas C., Le Deist F., Cavazzana-Calvo M., Benkerrou M., Haddad E., Blanche S., Hartmann W., Friedrich W., Fischer A. (1995). Results of allogeneic bone marrow transplantation in patients with leukocyte adhesion deficiency. Blood.

[83] Yamazaki-Nakashimada M., Maravillas-Montero J.L., Berron-Ruiz L., Lopez-Ortega O., Ramirez-Alejo N., Acevedo-Ochoa E., Rivas-Larrauri F., Llamas-Guillen B., Blancas-Galicia L., Scheffler-Mendoza S. (2015). Successful adjunctive immunoglobulin treatment in patients affected by leukocyte adhesion deficiency type 1 (LAD-1). Immunol. Res..

[84] Moutsopoulos N.M., Zerbe C.S., Wild T., Dutzan N., Brenchley L., DiPasquale G., Uzel G., Axelrod K.C., Lisco A., Notarangelo L.D. (2017). Interleukin-12 and Interleukin-23 Blockade in Leukocyte Adhesion Deficiency Type 1. N. Engl. J. Med..

[85] Wang B., Lim J.H., Kajikawa T., Li X., Vallance B.A., Moutsopoulos N.M., Chavakis T., Hajishengallis G. (2019). Macrophage beta2-Integrins Regulate IL-22 by ILC3s and Protect from Lethal Citrobacter rodentium-Induced Colitis. Cell Rep..

[86] Marsili M., Lougaris V., Lucantoni M., Di Marzio D., Baronio M., Vitali M., Lombardi G., Chiarelli F., Breda L. (2014). Successful anti-TNF-alpha treatment in a girl with LAD-1 disease and autoimmune manifestations. J. Clin. Immunol..

[87] Nakagawa M., Bondy G.P., Waisman D., Minshall D., Hogg J.C., van Eeden S.F. (1999). The effect of glucocorticoids on the expression of L-selectin on polymorphonuclear leukocyte. Blood.

[88] Ballabh P., Kumari J., Krauss A.N., Shin J.J., Jain A., Auld P.A., Lesser M.L., Cunningham-Rundles S. (2003). Soluble E-selectin, soluble L-selectin and soluble ICAM-1 in bronchopulmonary dysplasia, and changes with dexamethasone. Pediatrics.

[89] Koehne P.S., Wagner M.H., Willam C., Sonntag J., Buhrer C., Obladen M. (2002). Soluble intercellular cell adhesion molecule-1 and L-selectin plasma concentrations and response to surfactant in preterm infants. Pediatr. Crit. Care Med..

[90] Lorant D.E., Li W., Tabatabaei N., Garver M.K., Albertine K.H. (1999). P-selectin expression by endothelial cells is decreased in neonatal rats and human premature infants. Blood.

[91] Pieh C., Kruger M., Lagreze W.A., Gimpel C., Buschbeck C., Zirrgiebel U., Agostini H.T. (2010). Plasma sE-selectin in premature infants: A possible surrogate marker of retinopathy of prematurity. Investig. Ophthalmol. Vis. Sci..

[92] Zielinska K.A., Van Moortel L., Opdenakker G., De Bosscher K., Van den Steen P.E. (2016). Endothelial Response to Glucocorticoids in Inflammatory Diseases. Front. Immunol..

[93] Nagaraj R., Stack T., Yi S., Mathew B., Shull K.R., Scott E.A., Mathew M.T., Bijukumar D.R. (2020). High Density Display of an Anti-Angiogenic Peptide on Micelle Surfaces Enhances Their Inhibition of alphavbeta3 Integrin-Mediated Neovascularization In Vitro. Nanomaterials.

[94] Ley K., Rivera-Nieves J., Sandborn W.J., Shattil S. (2016). Integrin-based therapeutics: Biological basis, clinical use and new drugs. Nat. Rev. Drug Discov..

[95] Frenette P.S. (2001). Locking a leukocyte integrin with statins. N. Engl. J. Med..

[96] Turunen R., Nupponen I., Siitonen S., Repo H., Andersson S. (2006). Onset of mechanical ventilation is associated with rapid activation of circulating phagocytes in preterm infants. Pediatrics.

[97] Desai L.P., Sinclair S.E., Chapman K.E., Hassid A., Waters C.M. (2007). High tidal volume mechanical ventilation with hyperoxia alters alveolar type II cell adhesion. Am. J. Physiol. Lung Cell. Mol. Physiol..

